# Hybrid Faster R-CNN for Tooth Numbering in Periapical Radiographs Based on Fédération Dentaire Internationale System

**DOI:** 10.3390/diagnostics15222900

**Published:** 2025-11-15

**Authors:** Yong-Shao Su, I Elizabeth Cha, Yi-Cheng Mao, Li-Hsin Chang, Zi-Chun Kao, Shun-Yuan Tien, Yuan-Jin Lin, Shih-Lun Chen, Kuo-Chen Li, Patricia Angela R. Abu

**Affiliations:** 1 Division of Periodontics, Department of Dentistry, Taoyuan Chang Gung Memorial Hospital, Taoyuan City 33305, Taiwan; mr2053@cgmh.org.tw; 2 Undergraduate Program in Intelligent Computing and Big Data, Chung Yuan Christian University, Taoyuan City 32023, Taiwan; 3 Department of Operative Dentistry, Taoyuan Chang Gang Memorial Hospital, Taoyuan City 33305, Taiwan; louiszzzzz@cgmh.org.tw; 4 Department of Electronic Engineering, Chung Yuan Christian University, Taoyuan City 32023, Taiwan; s11226145@cycu.edu.tw (L.-H.C.); s11226231@cycu.edu.tw (Z.-C.K.); s11226210@cycu.edu.tw (S.-Y.T.); 5 Department of Program on Semiconductor Manufacturing Technology, Academy of Innovative Semiconductor and Sustainable Manufacturing, National Cheng Kung University, Tainan City 70101, Taiwan; m28121562@gs.ncku.edu.tw; 6 Department of Information Management, Chung Yuan Christian University, Taoyuan City 32023, Taiwan; kuochen@cycu.edu.tw; 7 Ateneo Laboratory for Intelligent Visual Environments, Department of Information Systems and Computer Science, Ateneo de Manila University, Quezon City 1108, Philippines; pabu@ateneo.edu

**Keywords:** automated dental diagnostic, Faster R-CNN, Fédération Dentaire Internationale System, periapical radiographs, tooth-numbering localization

## Abstract

**Background/Objectives:** Tooth numbering is essential because it allows dental clinicians to identify lesion locations during diagnosis, typically using the Fédération Dentaire Internationale system. However, accurate tooth numbering is challenging due to variations in periapical radiograph (PA) angles. In this study, we aimed to develop a deep learning-based tool to assist dentists in accurately identifying teeth via tooth numbering and improve diagnostic efficiency and accuracy. **Methods:** We developed a Hybrid Faster Region-based Convolutional Neural Network (R-CNN) technique and a custom loss function tailored for PA tooth numbering to accelerate training. Additionally, we developed a tooth-numbering position auxiliary localization algorithm to address challenges associated with missing teeth and extensive crown loss in existing datasets. **Results:** We achieved a maximum precision of 95.16% utilizing the transformer-based NextViT-Faster R-CNN hybrid model, along with an accuracy increase of at least 8.5% and a 19.8% reduction in training time compared to models without the proposed tooth-numbering position auxiliary localization algorithm and conventional methods. **Conclusions:** The results demonstrate the effectiveness of the proposed method in overcoming challenges in PA tooth numbering within AI-assisted dental diagnostics, enhancing clinical efficiency, and reducing the risk of misdiagnosis in dental practices.

## 1. Introduction

Oral health issues are increasingly becoming a public concern worldwide, with more and more people visiting medical clinics for oral examinations and dental care, increasing the workload of dental professionals [1,2]. Typically, people visit dental clinics for various procedures, such as caries treatment [3], orthodontic treatment [4], dental scaling [5], cosmetic veneers [6], oral ulcer management [7], and other treatments. Furthermore, the prevalence of oral diseases has been steadily rising over the years, coupled with an aging population that has driven a growing demand for dental care, resulting in a continuous increase in the workload for dentists [8]. In this context, effectively managing patients, reducing waiting times, and enhancing diagnostic efficiency have become significant challenges in dental clinic management.

In traditional dental clinical practice, dentists typically perform preliminary clinical diagnoses using X-ray images [9]. Standard imaging techniques in this regard include Dental Panoramic Radiographs (DPRs) and Periapical Radiographs (PAs). A DPR provides a comprehensive view of the entire oral structure, allowing for the observation of both the maxillary and mandibular arches and the alignment of the teeth [10]. In contrast, a PA targets specific teeth and their apical regions, clearly displaying the root and surrounding bone changes, making it particularly suitable for diagnosing caries, apical lesions, and periodontal diseases [11]. After the imaging examination, dentists mark the affected teeth according to the Fédération Dentaire Internationale (FDI) tooth-numbering system [12]. The FDI system uses a two-digit code, where the first digit indicates the quadrant, and the second digit specifies the tooth number within that quadrant. Although this coding system is internationally consistent in facilitating cross-border diagnosis and communication, practical challenges still exist. In fast-paced clinical environments or when multiple dentists collaborate, errors may arise due to memory-related confusion or writing mistakes, leading to incorrect tooth numbering and an increased risk of diagnostic errors. This issue becomes even more pronounced in cases involving complex oral conditions or multiple lesions [13].

An increasing number of medical institutions have begun to adopt intelligent dental systems to address the aforementioned challenges, such as artificial intelligence (AI)-assisted imaging diagnostic techniques [14,15,16,17,18], which enable early detection of implants [19], implant brand identification [20], and periodontal disease detection [21]. In developing AI applications for dental image diagnosis, significant progress has been made in automating FDI tooth numbering using DPRs. Several studies have demonstrated that deep learning-based AI algorithms can accurately identify tooth positions and numbers in a DPR, effectively reducing the errors associated with traditional manual labeling. For instance, Adnan et al. [22] utilized a U-Net combined with Faster R-CNN for DPR-based tooth numbering, achieving a precision of 88.8% and a recall of 87.3%. Ayhan et al. [23] developed an improved CNN model for detecting tooth numbers, achieving an accuracy of 93.4% and a recall of 83.4%. Ali et al. [24] also made a significant breakthrough in DPR-based tooth numbering using a computer vision model, namely, the seventh version of You Only Look Once (YOLOv7, Academia Sinica, Taiwan, released in 2022), achieving an average accuracy of 98.2%.

However, compared to the well-established application of AI in FDI tooth numbering using DPRs, there remains substantial room for improvement in AI-assisted diagnosis with PAs. Although a PA can display the root and surrounding bone conditions and is clinically valuable for diagnosing apical lesions and periodontal problems, its imaging characteristics are highly complex [25]. Due to the narrow field of view, a PA is prone to tooth overlapping, angle variations, and anatomical structure differences. This leads to deviations in AI-based tooth position recognition and lesion marking. Despite some attempts to apply CNNs for automatic tooth numbering in PAs, the high complexity of data annotation and variability among cases make it challenging for models to achieve clinical-grade accuracy. For example, Görürgöz et al. [26] utilized an R-CNN to automatically detect and number teeth in periapical images, achieving a precision of approximately 78.72%. Moreover, Chen et al. [27] used Mask R-CNN for tooth position detection, obtaining an accuracy of 88.8%. Furthermore, current AI-based PA tooth-positioning studies often exclude PAs featuring implants, missing teeth, or extensive crown damage, as these cases may introduce significant errors in model validation [26,28].

In summary, there are still numerous challenges in applying PAs for localizing teeth based on tooth numbering. There are also ethical issues [15], including whether the interpretation results from AI-assisted diagnostic software could mislead clinicians. Thus, it is important and necessary to design a tool with careful validation and confirmation, especially if this method can directly support dentists in reducing diagnostic errors and fatigue. To assist dentists in clinical diagnosis and mitigate the risk of misrecording lesion locations due to fatigue from prolonged work, potentially leading to delayed treatment or misdiagnosis, we developed an innovative auxiliary tooth-numbering technique compatible with the FDI tooth-numbering system for PAs and successfully identified the missing teeth in the dataset. The dataset utilized in this study includes PA with implants, missing teeth, and extensive crown damage, ensuring model generalizability and addressing the limitations of previous research that excluded such cases. Moreover, we designed a novel tooth-numbering position loss function that significantly reduces the training time required for deep learning models, minimizing the time needed to update the database. Additionally, we developed an advanced tooth-positioning assistance algorithm capable of accurately identifying tooth numbers. This innovation enhances the efficiency of clinical diagnosis for dentists and effectively reduces diagnostic errors, ultimately improving the quality of dental care.

## 2. Materials and Methods

**Figure 1:**
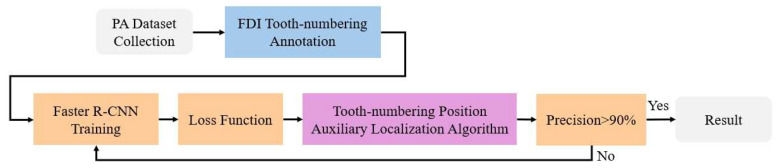
PA FDI tooth-numbering assistance flow chart.

This section introduces the detailed process of FDI tooth numbering based on PA. Figure 1 illustrates the research workflow. Firstly, we collected PAs from Chang Gung Memorial Hospital and annotated the ground-truth FDI tooth-numbering scheme. Subsequently, we applied the tooth-numbering position auxiliary localization algorithm and the customized loss function developed in this study. These components significantly reduced the training time of the deep learning model while maintaining high accuracy in FDI tooth numbering, even within a PA dataset containing implants, missing teeth, and extensive crown damage.

### 2.1. PA Dataset Collection and FDI Tooth-Numbering Annotation

This subsection introduces the dataset, the annotation methods, and the FDI tooth-numbering system utilized in this study to ensure the rigor and completeness of the research.

#### 2.1.1. PA Dataset Collection

The dataset used was provided by Chang Gung Memorial Hospital in Taoyuan, Taiwan. The medical center collected PAs from seven medical institutions across Taiwan. This study was approved by the Institutional Review Board (IRB) of Chang Gung Medical Foundation (IRB number: 202500009B0). The PAs and ground-truth annotations were collected by three experienced oral specialists, each with over five years of clinical practice. The criteria for the PAs excluded patients with a positive history of Human Immunodeficiency Virus (HIV) infection to avoid potential confounding factors. All eligible PAs from adult patients (Age ≥ 20) who met the inclusion criteria during the data collection period were incorporated into the dataset to maximize the sample size and ensure the generalizability of the clinical diagnosis. This study’s PA database contains 422 PAs. We divided the dataset into a training set with 254 PAs (75%) and a validation set with 70 PAs (25%), while retaining 98 additional PAs as a test set for evaluating model stability, listed in Table 1.

**Table 1:** Data classification for periapical images after preprocessing.

The Number of Datasets			
	**Training Set**	**Validation Set**	**Test Set**
Quantity	254	70	98

#### 2.1.2. FDI Tooth-Numbering System

**Figure 2:**
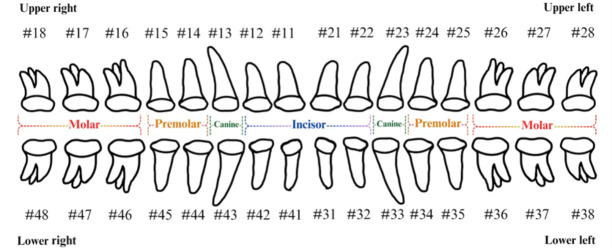
FDI tooth-numbering system.

The FDI tooth-numbering system is a widely used dental standard in clinical and academic settings across Europe and Asia. In this system, a two-digit code is employed to identify tooth positions: The first digit indicates the quadrant of the dental arch. Numbers 1 to 4 represent permanent teeth, arranged in a clockwise manner starting from the upper-right quadrant (1), followed by the upper left (2), the lower left (3), and the lower right (4). The second digit specifies the tooth number within the quadrant, starting from the central incisor and moving backward. For example, as shown in Figure 2, the second digit 1 and 2 represent the incisor teeth. Thus, #11 and #12 represent the upper right central incisors, #21 and #22 represent the upper left incisors, #31 and #32 represent the lower left incisors, and #41 and #42 represent the lower right incisors. The second digit 3 represents canine. The second digit 4 and 5 represent premolar. The second digit 6, 7, and 8 represent molar. Thus, based on the combination of two digits in FDI tooth-numbering system, the tooth position can be identified as incisor, canine, premolar, or molar located in specific quadrant(s).

#### 2.1.3. Tooth-Numbering Annotation

**Figure 3:**
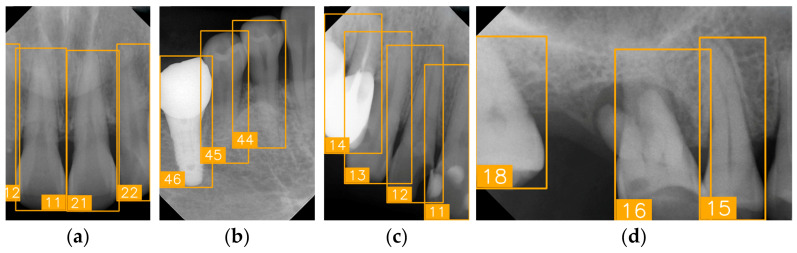
PA tooth-numbering annotation. (**a**) Normal tooth. (**b**) Implant. (**c**) Extensive crown loss. (**d**) Missing tooth.

LabelImg (version 1.7.0, https://github.com/HumanSignal/labelImg (accessed on 9 November 2025), U.S.A., released in 2013) was used for tooth-numbering annotation. The PA annotation process was conducted under three senior supervisors’ supervision. The annotation focused on labeling all visible teeth within the images, using square bounding boxes to mark each tooth. The annotations covered the complete position of each tooth, from the apex to the crown. All tooth numbers followed the FDI two-digit system, as shown in Figure 2. During the annotation process, each annotator worked independently, and the final annotations were thoroughly reviewed and validated by the three senior supervisors to ensure completeness and reliability, as shown in Figure 3. In (a), the teeth are normal incisors in the upper two quadrants. (b) indicated the teeth are implant premolars and the first molar on the lower right. As (c) represented the extensive crown loss on the upper right, including two incisors, one canine, and the first premolar. (d) successfully positioned the second tooth of the premolar, the first and third teeth of the molar, and pointed out the missing tooth in the upper right.

### 2.2. Tooth-Numbering Position Loss Function

Accurate tooth position localization is crucial for clinical diagnosis and treatment planning in PA lesion analysis performed by dentists. However, due to variations in radiographic angles, tooth occlusions, missing teeth, or implants, most existing studies exclude such datasets, limiting the performance of models in executing FDI tooth-numbering tasks. We propose a method based on Kullback–Leibler Divergence Loss (KL DivLoss) designed to optimize spatial relationships in tooth-numbering position classification. By leveraging a soft label mechanism, the approach enhances the accuracy of tooth-numbering localization and improves training efficiency.

#### 2.2.1. Hard Label and Soft Label

Hard Label and Soft Label are two standard methods for representing labels. Hard Label is a discrete label that typically uses 0 or 1 to indicate a specific class, making it suitable for traditional supervised learning and classification tasks due to its simplicity and intuitive nature. However, in the context of tooth-numbering detection, the Hard Label method is overly rigid and fails to capture spatial proximity uncertainty. This limitation becomes particularly evident when the shooting angle of a PA makes it challenging to determine tooth numbers, leading to potential overfitting. In contrast, a Soft Label is a probabilistic label representing the probability distribution of a sample belonging to different classes, reflecting the model’s confidence in each class. This characteristic makes it more suitable for handling PA cases, including various adult tooth numbers.

From a practical design perspective, unlike a Hard Label, which assigns a probability of 1 to the actual class only, a Soft Label assigns non-zero probabilities to anatomically adjacent tooth positions. This representation reflects the anatomical relationship between teeth, effectively reducing severe misclassification errors (such as crossing the oral midline or including missing teeth). This approach allows a model to maintain tolerance between adjacent tooth positions, promoting smooth categorical distribution learning. Moreover, the soft probability distribution in Soft Label reduces loss oscillation during the early stages of training, accelerating model convergence. This property is particularly advantageous in clinical scenarios with limited training data or image quality. By assigning probabilities to neighboring tooth positions, Soft Label effectively handles complex situations involving missing teeth or implants. This enables a model to infer the correct numbering based on the spatial logic of the surrounding teeth, enhancing the robustness of clinical applications. In cases where images are blurry or boundaries are unclear, Soft Label can better express a model’s uncertainty and provide a confidence ratio for the area of different tooth positions.

#### 2.2.2. Kullback–Leibler Divergence Loss Function

Kullback–Leibler Divergence Loss (KL DivLoss) is a method used in machine learning and information theory to measure the difference between probability distributions. It is widely applied in deep learning tasks that involve probabilistic outputs or aligning distributions. KL DivLoss leverages Soft Label to capture spatial relationships, enabling precise modeling of the spatial characteristics of tooth positions. This approach significantly improves the performance of deep learning models in FDI tooth numbering. The total loss used in this study comprises the base loss and KL DivLoss. The base loss originates from the deep learning detection model, covering classification loss, bounding box regression loss, object detection loss, and RPN bounding-box loss, while KL DivLoss consists of a soft label probability distribution and the Kullback–Leibler divergence. During model training, a soft label distribution is generated for each ground-truth tooth label, assigning decreasing probability weights based on the spatial distance between tooth positions. For example, if the ground-truth label is #11, the neighboring positions #12 and #21 receive relatively higher probability weights, while more distant tooth positions (such as #18 or #28) have weights close to zero. The formulas for total loss and KL DivLoss are shown in (1) and (2), where P_soft_(i) represents the soft label probability distribution based on tooth spatial relationships, and P_pred_(i) represents the model’s predicted probability distribution.

(1) Total loss=Base loss+α∗KLDivLoss

(2) $$DivLoss=\sum_{i}P_{soft}(i)log(\frac{P_{soft}(i)}{P_{pred}(i)})$$

### 2.3. Tooth-Numbering Position Deep Learning and Parameter Settings

This subsection will introduce the deep learning model used for FDI tooth numbering in PAs, along with the relevant model parameters, detailed training environment settings, and architectural descriptions.

#### 2.3.1. PA Tooth-Numbering Deep Learning Model

Faster R-CNN and You Only Look Once (YOLO) are currently two of the most commonly used object detection techniques in dental medicine. Compared to YOLO, Faster R-CNN offers higher detection accuracy and more precise bounding box localization, especially when dealing with FDI tooth numbering, which involves objects of varying scales and overlapping structures. This advantage stems from the use of an RPN, which generates high-quality candidate regions, followed by Bounding Box Regression, which refines the classification and regression branches for precise localization and tooth numbering. To ensure the model adapts to the characteristics of PAs, we used Faster R-CNN, which is particularly effective when handling class imbalance in images (such as missing teeth or implant treatments), demonstrating greater stability. However, Faster R-CNN’s training and inference speed are relatively slower compared to YOLO, making it less suitable for real-time detection tasks. We utilized KL DivLoss to accelerate training and reduce the risk of overfitting to address this limitation.

Faster R-CNN was adopted as the primary detection framework in this study, and multiple backbones with spatial awareness and efficient feature extraction capabilities were introduced for comparison and experimentation. These backbones include classical CNN-based architectures such as ResNet50-FPN, MobileNet_v3, and MobileOne-S1. Additionally, we evaluated the performance of transformer-based architectures, including ConvNeXt_v2, MobileViT_v2-0.5, and NextViT, to assess their effectiveness in PA tooth-number detection. Regarding the characteristics of each backbone, ResNet50-FPN is a classical CNN-based residual network known for its stable performance and strong feature abstraction capabilities. MobileNet_v3 and MobileOne-S1 are lightweight architectures designed for faster inference speed and a lower parameter count, making them suitable for edge devices and real-time diagnostic scenarios. ConvNeXt_v2 is a recently proposed transformer-based modern convolutional architecture with a deeper and more expansive receptive field, ideal for handling anatomical variations in medical images. MobileViTv2-0.5 combines the mobile architecture with vision transformer components, enabling cross-regional correlation modeling. NextViT integrates local convolution and global attention modules, enhancing perception and compensation for non-standard arrangements or missing regions, making it particularly useful in scenarios involving root fractures or implant interference. Table 2 shows the architecture of the FDI tooth-numbering detection model.

#### 2.3.2. Parameter Setting and Experiment Platform

The tooth-number detection system proposed in this study is based on an object detection model that localizes tooth-number positions in PAs. We conducted experiments using Faster R-CNN as the base model. We also performed hyperparameter tuning to ensure consistent performance comparison, as shown in Table 3. The initial learning rate was 0.0001, the number of epochs was 50, and the batch size was 2.

#### 2.3.3. Model Training and Validation

After training was complete, we adopted multiple metrics to comprehensively evaluate the model’s performance, as shown in (3)–(9). These metrics reflect the model’s predictive capability in tooth numbering from various perspectives, including the exacting accuracy for different tooth positions and the model’s overall performance in practical applications. To further assess the model’s understanding of the spatial structure of tooth positions, we designed two custom metrics: FDI_error_ and TOP1_conf_. We set an IoU ≥ 0.5 as the threshold to filter predictions with accurate spatial localization, excluding results with significant localization errors. FDI_error_ measures the mean absolute difference between the predicted tooth position and the ground-truth index. A lower value indicates that the model has learned the spatial structure of tooth-number positions more accurately, maintaining reasonable spatial continuity even if the classification is incorrect. TOP1_conf_ represents the average confidence score when the model makes correct predictions (IoU ≥ 0.5 and a consistent category). A higher value indicates the model has greater confidence in making correct predictions, demonstrating its stability and reliability. We conducted extensive ablation studies and comparative experiments using the proposed loss function and the tooth-positioning assistance algorithm. The experimental results show the proposed technique exhibits significant advantages in performance.

(3) $$Precision=\frac{TP}{TP+FP}$$

(4) $$Specificity=\frac{TN}{TN+FP}$$

(5) $$Recall(Sensitivty)=\frac{TP}{TP+FN}$$

(6) $$F1-score=2\times \frac{precision\times recall}{precision+recall}$$

(7) $$mAP50=\frac{1}{N}\sum_{i=1}^{N}{AP}_{i}^{IoU=0.5}$$

(8) $${FDI}_{error}=\frac{1}{N}\sum_{i=1}^{N}\left|{FDI}_{pred,i}-{FDI}_{true,i}\right|$$

(9) $${TOP1}_{conf}=\frac{1}{N_{TP}}\sum_{i=1}^{N_{Tp}}pred\_scores[P_{idx},\;i]$$

### 2.4. Tooth-Number Position Auxiliary Localization Algorithm

After the model was trained, we integrated the tooth-number position auxiliary localization algorithm developed in this study. This algorithm’s primary goal is to address tooth-numbering errors caused by missing teeth or extensive crown defects commonly found in PA datasets. As shown in Algorithm 1, it first divided the adult teeth into two groups based on the upper and lower dental arches, storing the calculated number of teeth in variables U and L. Next, it slid the ideal tooth-position-numbering template along the sequence of model detection results and computed the maximum overlap to identify the initial index with the highest match count (M). If M > 1, the algorithm corrects the erroneous tooth numbering and outputs the accurate position; otherwise, it outputs the existing tooth numbering scheme. In cases where the comparison indicates misalignment, the algorithm automatically adjusts the tooth numbering based on the optimal alignment position, ensuring the final sequence aligns with the regular anatomical order.
**Algorithm 1.** Tooth-numbering position auxiliary localization algorithm.Input:
${p=[p}_{1},p_{2},p_{3}\ldots p_{n},P_{i}\in \left\{1,2,\ldots ,32\right\}$ Output:
$p^{\prime }=[p_{1}^{\prime },p_{2}^{\prime },\ldots ,p_{n}^{\prime }]$1:
$U=\sum_{i=1}^{n}\delta \left(p_{i}\leq 16\right),$ L
$=\sum_{i=1}^{n}\delta \left(p_{i}>16\right),\delta \left(condition\right)=\left\{\begin{array}{c} 1,if\;condition\;is\;True\\ 0,otherwise \end{array}\right.$ 2:
$A=\left\{\begin{array}{c} A_{u},if\;U>L\\ A_{l},otherwise \end{array}\right.,where\;A_{u}=\left[1,2,\ldots ,16\right]\;and\;A_{l}=\left[17,18,\ldots ,32\right]$ 3:
${Index=Argmax}_{i=0}^{\left|A\right|-n}\sum_{j=0}^{n-1}\delta (p_{j+1}=A_{i+j+1}$) 4:
$M=max(\sum_{j=0}^{n-1}\delta (p_{j+1}=A_{i+j+1}$)) 5:
$M=\left\{\begin{array}{c} \mathrm{if}\;>1,algorithm\;modify\\ \mathrm{if}\leq 1,\mathrm{no}\;changes \end{array}\right.$ 6:
${\;\;p}_{i}^{\prime }=\left\{\begin{array}{c} A_{index+i},\mathrm{if}\;p_{i}\neq A_{index+i}\;\mathrm{and}\;p_{i}>0\\ p_{i},\mathrm{otherwise} \end{array}\right.$

Most existing automated PA tooth-numbering systems tend to overlook the localization of missing teeth. In this study, we proposed a missing-tooth detection mechanism that can automatically identify the positions of potential missing teeth within the detection results. In the detection process, the mechanism first sorts the bounding boxes based on the horizontal coordinate (x_min_) from left to right. Then, it calculates the distance between adjacent teeth. If the gap exceeds a preset threshold, the algorithm determines that a tooth is missing at that position. To maintain positional integrity in the tooth-numbering sequence, the algorithm inserts a (0) as a placeholder at the identified missing-tooth position, facilitating subsequent tooth-numbering correction. This algorithm integrates Faster R-CNN for tooth detection, sequence alignment, missing-tooth detection, and result visualization, forming a comprehensive and efficient automated tooth position analysis solution. It can automatically correct misaligned tooth positions in the detection results and fill in the tooth numbering for missing teeth, providing more accurate and detailed results for clinical dental diagnosis.

## 3. Results

This section is divided into three subsections. The first subsection shows the baseline accuracy of Faster R-CNN with different backbones. The second subsection combines the loss function we designed and the tooth-numbering position auxiliary localization algorithm for PA tooth number localization. The third subsection will evaluate the technique developed in this study through clinical diagnostic trials, utilizing an open-source dataset to verify the reliability and generalizability of the proposed method in real-world medical applications.

### 3.1. Faster R-CNN Training Results

**Figure 4:**
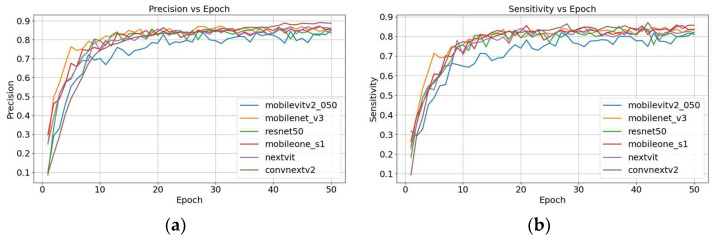
Hybrid Faster R-CNN’s training accuracy and training loss with different backbones: (**a**) Precision and (**b**) Sensitivity.

In this study, we first explored the tooth-numbering localization capability of Faster R-CNN using different backbones. Then, we selected CNN-based architectures, such as ResNet50, MobileNet_v3, and MobileOne_s1, as well as Transformer-based architectures, including ConvNeXt_v2, MobileViT_v2-0.5, and NextViT. As shown in Figure 4, ConvNeXt_v2 demonstrates significant superiority across most evaluation metrics, especially in Precision (87.29%), Specificity (99.44%), and mAP50 (80.11%), indicating its excellent performance in accurately localizing tooth numbers. NextViT also shows robust performance, particularly in Sensitivity (84.94%) and F1-score (85.77%), ranking second only to ConvNeXt_v2. This indicates that NextViT is well-suited to handling highly variable dental images. In contrast, the traditional CNN-based ResNet50 backbone exhibits weaker performance in Sensitivity (79.59%) and F1-score (81.77%), indicating its limitations in handling detailed variations and complex tooth structures. While MobileNet_v3 and MobileOne_s1 offer advantages in speed and efficiency, their overall performance remains moderate, particularly in terms of mAP50, with values of 76.21% and 78.71%, respectively. The MobileViT_v2-0.5 backbone performs well in Specificity (99.25%), but its lower performance in Sensitivity (79.71%) and F1-score (81.01%) suggests challenges when dealing with heterogeneous tooth positions. Figure 4 illustrates the training processes of different backbones, including with respect to the evolution of Precision and Sensitivity over time, highlighting each architecture’s comparative strengths and weaknesses in the context of tooth-numbering localization.

We further compared our approach with the current mainstream detection models, YOLOv8 (Ultralytics, Frederick, MD, U.S.A., released in 2023) [29] and YOLOv11 (Ultralytics, Frederick, MD, U.S.A., released in 2024) [30], as shown in Table 4. Faster R-CNN significantly outperformed YOLO models across all five-evaluation metrics. This result indicates that Faster R-CNN is more effective in handling complex structures and multi-scale objects in dental images than YOLO. Specifically, Faster R-CNN excels when multiple teeth overlap or images are blurry, enabling precise tooth position localization. Although YOLO models offer a speed advantage, tooth-numbering localization requires higher accuracy and stability.

### 3.2. DivLoss Function and Tooth-Numbering Position Auxiliary Localization Algorithm Training Results

Table 5 presents the training results using different KL DivLoss α parameters. It can be observed that the overall model accuracy does not vary significantly across different α values, remaining within the range of 86% to 88%. When α = 0.7, the F1-score reaches 87.06%, Precision is 88.25%, and Sensitivity is 85.90%, almost equivalent to the performance when α = 0, indicating that this setting maintains accuracy without causing a noticeable performance drop. Regarding convergence speed, the number of epochs and training time are more noticeably affected by the choice of α. When α = 0.7, the model achieves optimal performance within 32 epochs, while other α settings generally require 38 to 42 epochs. Regarding training time, α = 0.7 has the shortest duration, at 4162.60 s, indicating that this value helps accelerate model convergence. From the TOP1_conf_ and FDI_error_ metrics, it is evident that α = 0.7 achieves a TOP1_conf_ of 90.34% and an FDI_error_ of only 0.3942, demonstrating a more balanced performance and stability compared to other configurations. This suggests that a suitable KL DivLoss weight can significantly shorten the training time while maintaining stable accuracy.

**Figure 5:**
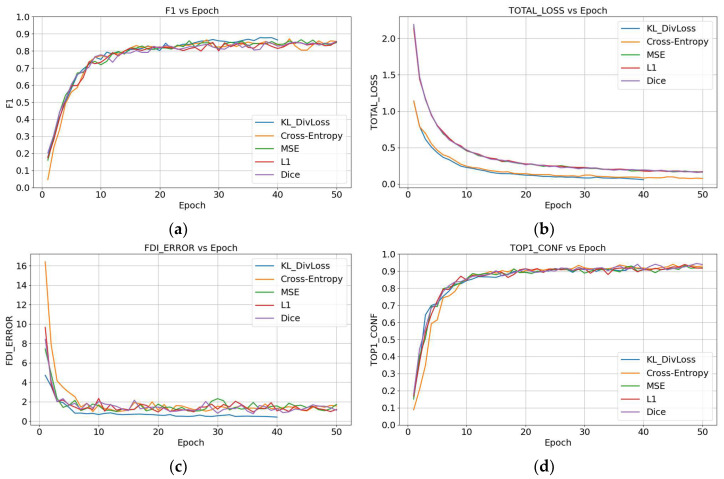
Training of Faster R-CNN using five different loss functions: (**a**) F1-score, (**b**) total loss, (**c**) FDI_error_, and (**d**) TOP1_conf_.

Figure 5a shows that KL DivLoss stabilizes after approximately 20 epochs, achieving a slightly higher F1-score than other loss functions, reaching around 0.86. In contrast, Cross-Entropy and MSE rapidly increase in the early stages but result in lower accuracy after convergence. Figure 5b plots total loss vs. epoch and shows that KL DivLoss decreases slowly in the initial stage but eventually demonstrates the best convergence performance, with a smooth and stable curve. Figure 5c shows the FDI_error_-vs.-epoch plot, indicating that KL DivLoss reaches the lowest error rate (around 0.4) after 20 epochs, significantly outperforming Cross-Entropy and MSE, which stabilize between 0.6 and 0.7. This indicates that KL DivLoss has a lower error rate in tooth-numbering localization, highlighting its superiority. Figure 5d shows the TOP1_conf_-vs.-Epoch graph, which indicates that KL DivLoss consistently maintains a confidence level of around 0.88 with minimal fluctuation, indicating high stability and reliability in the model’s prediction results. Overall, KL DivLoss exhibits significant advantages over other loss functions, particularly in enhancing feature learning and suppressing overconfidence.

**Figure 6:**
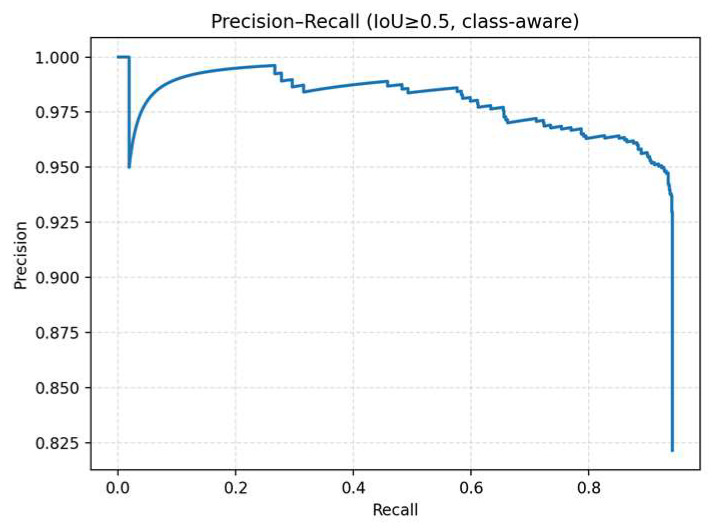
The Precision–recall curve under the condition of an IoU ≥ 0.5.

Figure 6 shows the Precision–recall curve, demonstrating that the model achieves excellent detection performance, with an IoU ≥ 0.5 (class-aware). The overall curve is smooth and well distributed, with precision remaining above 0.95 across most recall ranges. This indicates that the model maintains high accuracy even as it detects more targets. The initial segment of the curve rises nearly vertically, suggesting that the model produces almost no false positives at high confidence thresholds. Precision begins to decline slightly only after recall exceeds 0.9, which is a reasonable phenomenon in the high-recall regime. Based on the area under the curve, the estimated average precision (AP@0.5) is approximately 0.96–0.97, indicating that the model strikes a strong balance between sensitivity and specificity and offers stable, clinically applicable classification reliability.

Table 6 shows there are significant performance differences among the various models when the tooth-numbering localization assistance algorithm was used. All evaluations were conducted under the same IoU threshold (IoU ≥ 0.5). Relative to Table 7, which presents results without the algorithm, NextViT demonstrates the best performance, especially in Precision (95.16%), Sensitivity (94.60%), Specificity (99.82%), and F1-score (94.88%), achieving the highest levels across all these metrics. Additionally, NextViT shows an impressive mAP50 of 88.42%, indicating highly accurate and stable performance in the tooth-numbering localization task. ConvNeXt_v2 performs well in Precision (93.66%) and Specificity (99.72%), although its mAP50 is relatively lower, at 87.77%. In contrast, traditional ResNet50 and MobileNet_v3 exhibit relatively weaker performance in terms of Sensitivity and F1-score, with mAP50 values of only 83.85% and 83.32%. NextViT demonstrates the most outstanding comprehensive performance in the tooth-numbering localization assistance algorithm, particularly excelling in accuracy, stability, and inference efficiency. These characteristics make it highly suitable for practical clinical diagnostic applications.

**Figure 7:**
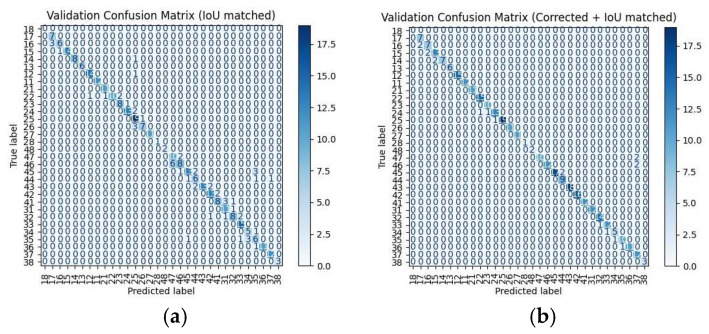
Confusion matrix comparison: (**a**) original and (**b**) algorithm modification.

Figure 7 presents the confusion matrix of the tooth-numbering predictions. It is evident from the figure that the improved confusion matrix exhibits more concentrated values along the diagonal axis, indicating that the model has become significantly more accurate in tooth-numbering prediction. In the original results shown in Figure 7a, the predicted labels for some tooth positions are more widely distributed, particularly in certain regions (such as positions 20 to 32), where prediction shifts are more pronounced. This indicates that the model originally had difficulty handling specific tooth positions, leading to confusion or misclassification. In the improved results shown in Figure 7b, the number of prediction errors in the problematic regions has been significantly reduced, with most values now concentrated along the diagonal line. According to the confusion matrix, there are two cases where a tooth number was misclassified as tooth numbers 47 and 46, respectively. From the perspective of spatial anatomy, teeth 37, 47, and 46 are all mandibular molars with similar biological characteristics, often leading to misclassification. This demonstrates that the improved algorithm effectively corrects the previous prediction biases, greatly enhancing the accuracy and stability of the model in the tooth-numbering recognition task.

**Figure 8:**
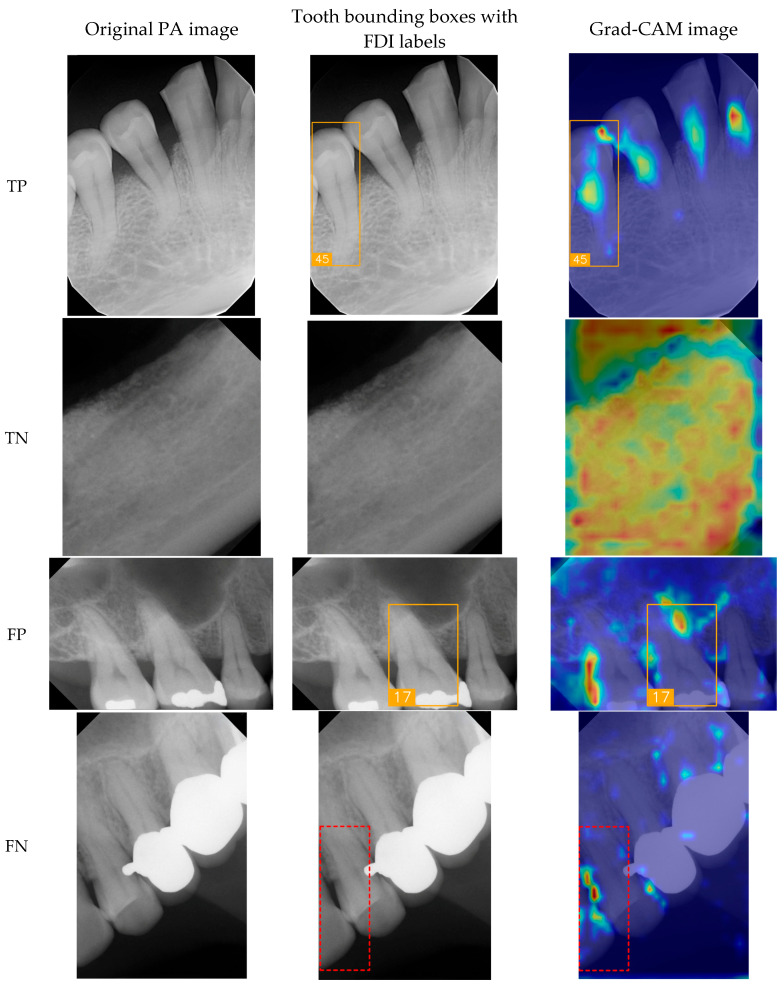
Case panels for TP, TN, FP, and FN for original PA images, tooth bounding boxes with FDI labels, and Grand-CAM images.

To show the cases for True Positives (TPs), True Negatives (TNs), False Positives (FPs), and False Negatives (FNs), a group of sample images was generated (Figure 8) for the original PA image, tooth bounding boxes with FDI labels, and Gradient-weighted Class Activation Mapping (Grad-CAM) images [31]. In the TP cases, the model effectively identified key dental features such as tooth contours and crown morphology, enabling accurate classification of FDI tooth numbers. Here, the proposed model successfully identified #45, the second tooth of the premolar located in the lower right, in tooth bounding boxes with FDI labels and Grad-CAM image. In the TN cases, validating its ability to distinguish background regions and prevent false-positive detections, the model demonstrated its capacity to correctly suppress misclassification in non-dental areas like bony structures and background noise, thereby ensuring the system’s clinical reliability. False detections arise from several factors; one common cause is the similarity of imaging features between adjacent teeth, where subtle differences are not easily distinguishable. As illustrated in Figure 8, the model misclassified tooth FDI number 16 as FDI number 17 because both are molars with similar morphological characteristics. Another cause is structural disruption due to dental implants, crowns, or fillings, which obscure the original anatomical features and lead to misclassification. The Grad-CAM visualizations indicate that the model focused on locally similar features but could not interpret the overall structural context. Missed detections can result from several factors, including tooth occlusion, insufficient image contrast, being positioned at the image boundary, or abnormal morphology that deviates from the patterns seen in the training data. In such cases, the model exhibits inadequate feature attention, leading to detection failure.

**Figure 9:**
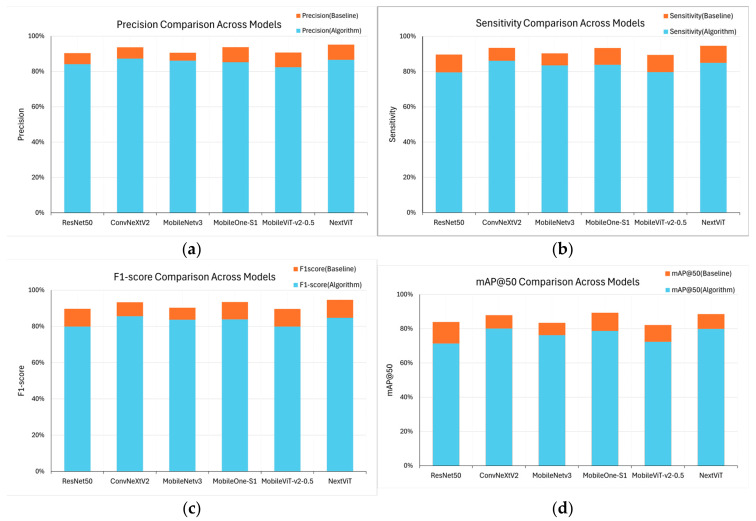
Comparison of the performance of different models before and after algorithm optimization: (**a**) Precision, (**b**) Sensitivity, (**c**) F1-score, and (**d**) mAP50.

Figure 9 compares four performance metrics across different models. The blue sections represent the original baseline performance, while the orange sections indicate performance improvement after the proposed algorithm was integrated. It is evident that after the algorithm was introduced, all the models exhibited varying degrees of enhancement across all metrics. The improvement is particularly notable in the case of NextViT and MobileOne_s1. NextViT achieved a score of over 94% in Precision, Recall, and F1-score, while mAP50 increased from the baseline 83% to 88%, indicating its superior accuracy and stability in handling tooth-numbering localization tasks. In contrast, the traditional ResNet50 also showed improvement in Precision and Recall, but this enhancement is relatively minor, reflecting inherent structural limitations that hinder the algorithm’s full potential. Moreover, ConvNeXt_v2 and MobileOne_s1 showed relatively consistent performance improvements after algorithm optimization, especially in F1-score and mAP50, indicating better generalization capabilities.

### 3.3. Clinical FDI Tooth-Number Detection Results

**Figure 10:**
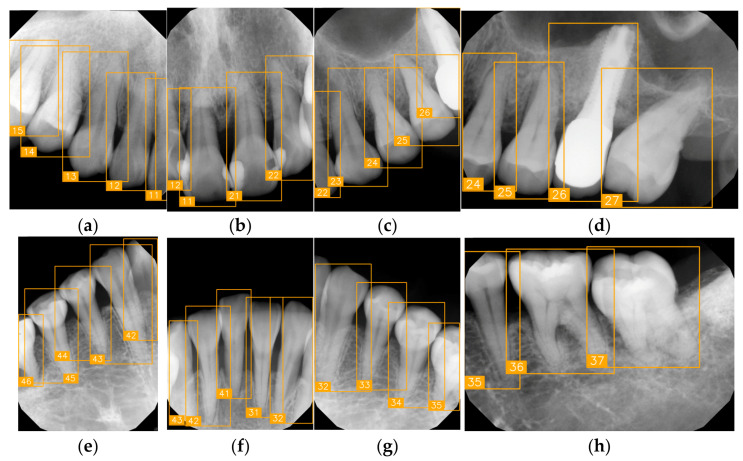
Multi-angle PA of the same patient for tooth-numbering localization (**a**–**h**).

In this study, we verified tooth-number localization using various PAs from a clinical practice. Figure 10 shows a set of PAs taken from a patient during clinical diagnosis with various angles, and eight of them were chosen, as labeled from (a) to (h). This PA set nearly covers the entire upper and lower jaws, featuring multiple teeth. Despite certain images exhibiting low quality or lacking prominent anatomical landmarks due to angle differences, the proposed algorithm accurately marked each tooth-numbering position with original bounding boxes and labeled the corresponding FDI numbers, facilitating tooth position identification and subsequent diagnosis. We observed that the arrangement of teeth and image quality varied across different PAs. For example, Figure 10a,e show precise tooth edges and structures, while Figure 10d,h show misidentification due to apical lesions or blurry images. Despite these quality issues, the tooth-positioning assistance algorithm effectively performed numbering and annotation even under suboptimal imaging conditions. These experimental results demonstrate that the proposed tooth localization technique exhibits high stability and accuracy in clinical applications. It proves particularly useful when handling PAs captured under different conditions, effectively assisting dentists in quickly identifying tooth numbers, thereby reducing diagnostic pressure and minimizing the risk of misjudgment.

Table 8 presents several clinically challenging or hard-to-interpret PAs regarding tooth-number localization. These images pose significant challenges in terms of accurate tooth numbering due to their complex anatomical structures. We tested the proposed tooth-numbering assistance algorithm on these challenging images to verify its reliability when handling complex dental scenarios. The results demonstrate that the algorithm accurately labeled tooth positions in various challenging cases, including fractured teeth and implants (as shown in (a), (e), and (g)), tooth overlapping (as shown in (b) and (f)), missing teeth (as shown in (c)), and incomplete crowns (as shown in (d)). Most of the teeth were successfully identified, but (h), the implant image, was numbered incorrectly during tooth numbering owing to interference. After a slight adjustment, the result improved. This indicates that the algorithm excels in handling abnormal dental features and image-overlapping issues. Particularly in regions with root loss or implants, the model effectively identifies tooth positions by extracting residual features, thereby minimizing incorrect annotations. The proposed algorithm exhibited strong generalization ability and demonstrated clinical applicability when facing diverse imaging challenges, especially in cases involving tooth overlap, missing teeth, and dental implants. In addition, the NextViT model achieved a practical balance between diagnostic accuracy and computational efficiency, with an average inference time of 74.3 milliseconds per image. It delivered an F1-score of 85.77% and a specificity of 99.32%, indicating reliable performance in identifying relevant cases while minimizing False Positives. The results suggest that NextViT is well-suited for integration into PACS systems as a real-time diagnostic support tool and for batch processing in large-scale tuberculosis screening programs.

## 4. Discussion

The primary goal of this study was to utilize deep learning techniques for FDI tooth-number localization while integrating the proposed KL DivLoss function to accelerate the training of deep learning models, allowing them to achieve optimal stability and accuracy at an earlier stage. Additionally, we employed the tooth-numbering position auxiliary localization algorithm to address the challenges present in current research, including missing teeth, dental implants, low brightness, and extensive crown fractures in PAs. Compared with models in existing studies, the proposed Hybrid Faster R-CNN approach significantly outperforms previous state-of-the-art methods in PA tooth-number localization across most evaluation metrics. Specifically, it achieved a precision of 95.16%, a sensitivity of 94.60%, and an F1-score of 94.88%, indicating a highly accurate and balanced performance in identifying and localizing teeth. Compared to Görürgöz, C. et al.’s R-CNN baseline, which showed lower precision (78.72%) and a lower F1-score (87.20%) [26], the proposed method offers substantial improvements in both detection accuracy and consistency. Although Chen, C.-C. et al.’s U-Net-Mask-R-CNN model achieved a slightly higher mAP50 (90.73% vs. 88.42%) [27], its lack of reported sensitivity and F1-score limits a full comparison. Overall, the proposed approach demonstrated superior precision and robustness, suggesting its strong potential for clinical deployment in automated dental diagnostics.

Below are three key ways in which this study contributes to clinical auxiliary diagnosis.

- We proposed a Hybrid Faster R-CNN method to address challenges in dental position identification. Unlike other studies, the dataset used in this study includes common clinical scenarios, such as dental implants, incomplete crowns, and missing teeth. The proposed method achieved a maximum accuracy exceeding 95%, demonstrating its effectiveness in handling complex dental cases.
- During the model-training process, integrating the KL DivLoss function significantly accelerated model convergence, addressing the challenge posed by the increasing size of PA tooth position datasets, which makes it difficult for models to reach optimal convergence points. Compared to models without KL DivLoss, this approach reduces training time by 19.8%.
- After we incorporated the tooth-number position auxiliary localization algorithm, the accuracy of tooth-numbering localization improved by approximately 8.5%. The NextViT-Faster R-CNN hybrid model achieved the highest accuracy, reaching 95.16%, indicating its superior performance in dental position recognition.

However, this study still has some limitations. In the FDI system-based tooth classification task, the primary challenge relates to the insufficient size of the dataset. Although we utilized a dataset of 422 PAs, most of the collected data were from Asian populations. In future research, we plan to collaborate with institutions across different regions and utilize open-source PA databases to train the model, thereby enhancing its generalization capability and potentially further validating the method’s effectiveness in interpreting dental images across diverse ethnic groups. The training dataset is relatively small, so it may have introduced sampling bias and model instability or even optimistic variance in estimates. But even if the dataset is small, it could be useful in pretraining, self-supervised initiatives, and/or prospective data accrual for further extension.

The second limitation is the difficulty of the fine-grained classification of the 32 tooth positions using deep learning models. For example, positions 15 and 13 are often mistakenly identified as position 14 due to the high similarity of imaging features between adjacent teeth. The subtle anatomical differences between positions 13, 14, and 15 in X-ray images and factors such as shooting angle or tooth alignment may result in image overlap and blurring, making it easy for the model to misclassify these teeth. In the future, we will aim to improve the model’s performance in FDI tooth classification tasks by incorporating image enhancement techniques and developing PA-specific tooth localization models. The third limitation is that when more than two teeth are missing in the PA, the model may fail to accurately identify tooth positions due to insufficient relative positional and neighboring reference information.

This research has primarily focused on X-ray imaging. Studies involving CBCT and cephalometric imaging are still in the early stages and have only begun to emerge this year. Therefore, in future research, we will focus on scenarios with insufficient tooth numbers, aiming to develop a more robust spatial reasoning mechanism to help the model correctly infer tooth positions even when there is incomplete information, using a dataset including X-ray, CBCT, and cephalometric images. Moreover, future work will extend beyond accurate FDI tooth-number localization to address common lesion detection issues in PAs, thus helping to enhance diagnostic support in dental care.

## 5. Conclusions

The primary objective was to develop a deep learning-based method for PA-based tooth-number localization that can assist dentists in their clinical practices. This method addresses various practical clinical challenges that may lead to tooth position misjudgment, considering multiple dental conditions commonly encountered during diagnosis. These challenges are effectively addressed through the tooth-number position auxiliary localization algorithm developed in this study. Additionally, integrating the KL DivLoss function during model training ensures better convergence, enhancing the model’s stability and accuracy. The dataset is relatively small, but it could be useful in pretraining, self-supervised initiatives, and/or prospective data accrual for further extension. This study provides an innovative tooth localization technique that supports dentists in clinical PA-based diagnosis, contributing to the advancement of clinical dental care.

## Figures and Tables

**Table 2:** ConvNeXt_v2-Tiny-Faster R-CNN.

Block Number	Type	Kernel Size	Stride	Filters	Feature Map Size
0	Conv2D	4 × 4	4	96	56 × 56 × 96
1	Depthwise Conv + MLP	3 × 3	1	96	56 × 56 × 96
2	Depthwise Conv + MLP	3 × 3	1	96	56 × 56 × 96
3	Depthwise Conv + MLP	3 × 3	1	96	56 × 56 × 96
4	Downsampling Conv	2 × 2	2	192	28 × 28 × 192
5	Depthwise Conv + MLP	3 × 3	1	192	28 × 28 × 192
6	Depthwise Conv + MLP	3 × 3	1	192	28 × 28 × 192
7	Depthwise Conv + MLP	3 × 3	1	192	28 × 28 × 192
8	Downsampling Conv	2 × 2	2	384	14 × 14 × 384
9	Stage 3 Block (4 layers)	3 × 3	1	384	14 × 14 × 384
10	Stage 3 Block (4 layers)	3 × 3	1	384	14 × 14 × 384
11	Stage 3 Block (4 layers)	3 × 3	1	384	14 × 14 × 384
12	Stage 3 Block (4 layers)	3 × 3	1	384	14 × 14 × 384
13	Stage 3 Block (4 layers)	3 × 3	1	384	14 × 14 × 384
14	Downsampling Conv	2 × 2	2	768	7 × 7 × 768
15	Depthwise Conv + MLP	3 × 3	1	768	7 × 7 × 768
16	Depthwise Conv + MLP	3 × 3	1	768	7 × 7 × 768
17	Depthwise Conv + MLP	3 × 3	1	768	7 × 7 × 768
18	Global Average Pool	-	-	-	7 × 7 × 768
19	Fully Connected (FC)	-	-	32	1 × 1 × 32

**Table 3:** The hardware and software platforms and their versions.

Hardware Platform	Version
CPU	Intel Core i9-12900H
GPU	NVIDIA GeForce RTX 3070 Ti
DRAM	32 GB DDR5
**Software platform**	**Version**
Python	3.10.13
PyTorch	2.2.0
CUDA	12.1

**Table 4:** Comparison with YOLO architecture.

	Precision	Sensitivity	Specificity	F1-Score	mAP50	mAP @0.5:0.95
Faster R-CNN	87.29	86.16	99.44	86.72	80.11	50.80
	[85.1–89.5]	[83.9–88.4]	[98.9–99.9]	[84.5–88.9]	[77.5–82.7]	[46.8–54.8]
YOLOv8	49.38	57.90	NaN	53.27	38.56	22.54
	[45.6–53.1]	[54.1–61.7]		[49.4–57.1]	[35.1–42.1]	[20.6–24.5]
YOLOv11	54.02	58.82	NaN	56.30	60.57	36.54
	[50.2–57.8]	[55.0–62.5]		[52.6–60.0]	[57.2–63.9]	[33.3–39.7]

NaN: Not a Number

**Table 5:** Comparison with different KL DivLoss parameters.

α	Precision	Sensitivity	F1-Score	FDI_error_	TOP1_conf_	Epoch	Training Time
0	88.48%	86.49%	87.47%	1.373	91.00%	42	5193.79 s
0.2	87.06%	86.41%	86.73%	0.5073	89.19%	39	4129.63 s
0.5	88.23%	87.08%	87.66%	0.4557	90.55%	38	4203.73 s
0.7	88.25%	85.90%	87.06%	0.3942	87.98%	32	4162.60 s
1.0	86.80%	84.41%	85.59%	0.3155	88.70%	38	4390.91 s

**Table 6:** Comparison of different models integrating the tooth-numbering position auxiliary localization algorithm.

Algorithm	Precision	Sensitivity	Specificity	F1-Score	mAP50	mAP @0.5:0.95	*p*-Value
ResNet50	90.35	89.66	99.55	90.00	83.85	48.29	0.0967
ConvNext_v2	93.66	93.45	99.72	93.55	87.77	55.68	0.5867
MobileNet_v3	90.59	90.31	99.62	90.45	83.32	49.44	0.1358
Mobileone_s1	93.75	93.37	99.76	93.56	89.18	54.25	0.6092
MobileViT_v2-0.5	90.72	89.43	99.61	90.07	82.04	47.85	0.1222
NextViT	95.16	94.60	99.82	94.88	88.42	54.81	-

**Table 7:** Comparison with different backbones in Faster R-CNN training.

	Precision	Sensitivity	Specificity	F1-Score	mAP50	mAP @0.5:0.95
Resnet50	84.09	79.59	99.19	81.77	71.35	41.13
	[81.7–86.5]	[76.9–82.3]	[98.6–99.7]	[79.2–84.3]	[68.4–74.3]	[37.9–44.4]
ConvNext_v2	87.29	86.16	99.44	86.72	80.11	50.80
	[85.1–89.5]	[83.9–88.4]	[98.9–99.9]	[84.5–88.9]	[77.5–82.7]	[46.8–54.8]
MobileNet_v3	86.13	83.48	99.29	84.78	76.21	45.13
	[83.8–88.4]	[81.0–85.9]	[98.8–99.8]	[82.4–87.1]	[73.4–79.0]	[41.6–48.6]
Mobileone_s1	85.23	83.86	99.35	84.54	78.71	47.89
	[82.9–87.6]	[81.5–86.2]	[98.8–99.8]	[82.2–86.9]	[75.9–81.5]	[44.1–51.7]
MobileViT_v2-0.5	82.36	79.71	99.25	81.01	72.34	42.26
	[79.9–84.9]	[77.0–82.4]	[98.7–99.8]	[78.4–83.6]	[69.4–75.3]	[38.9–45.6]
NextViT	86.61	84.94	99.32	85.77	79.89	49.61
	[84.3–88.9]	[82.5–87.3]	[98.8–99.8]	[83.5–88.0]	[77.2–82.6]	[45.7–53.6]

**Table 8:** Evaluation of tooth-numbering localization in challenging dental radiographs.

Number	(a)	(b)	(c)	(d)
Condition	Fractured tooth; Implant	Overlaapping	Missing Tooth	Incomplete crown
PA	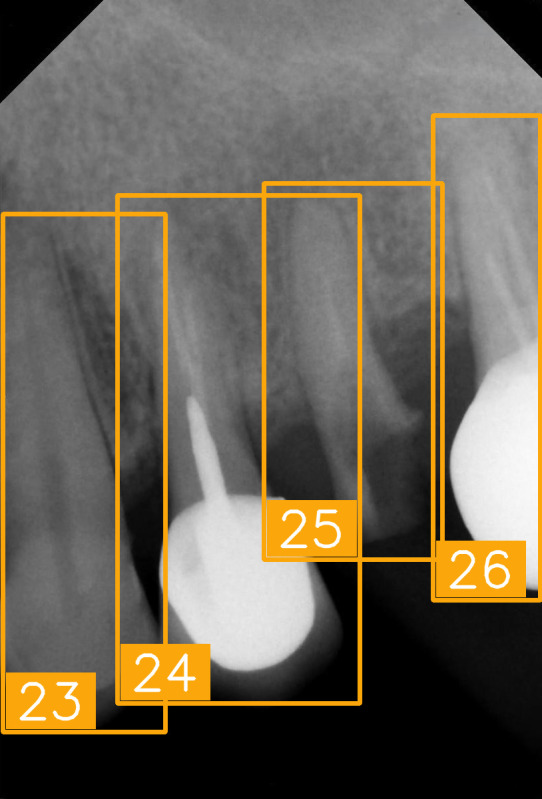	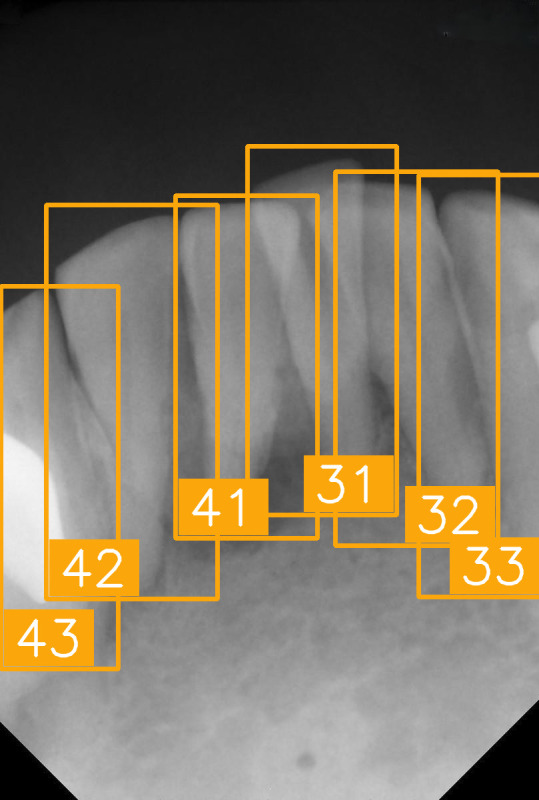	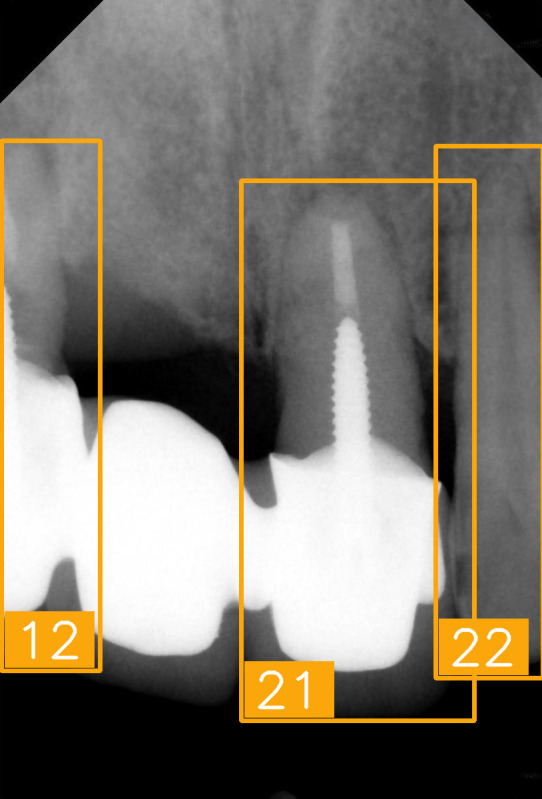	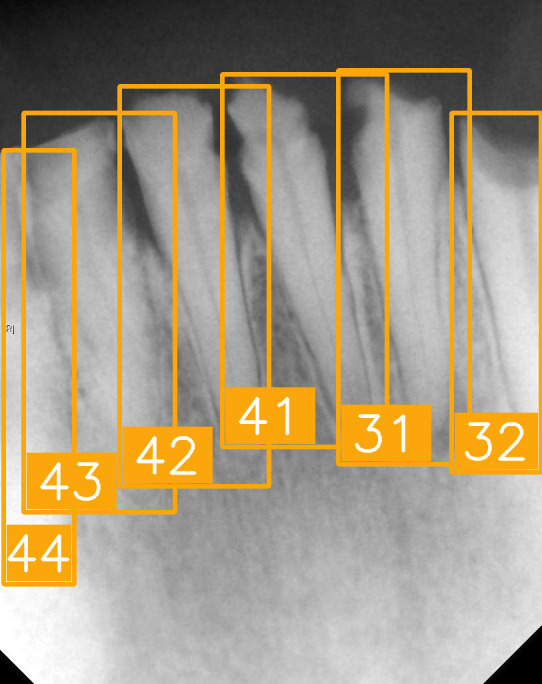
FDI ground truth number	23, 24, 25, 26	43, 42, 41, 31, 32, 33	12, 11, 21, 22	44, 43, 42, 41, 31, 32
Inference Time	75.9 ms	73.3 ms	76.1 ms	69.9 ms
Number	(e)	(f)	(g)	(h)
Condition	Fractured tooth; lower brightness	Overlapping	Implant	Implant
PA	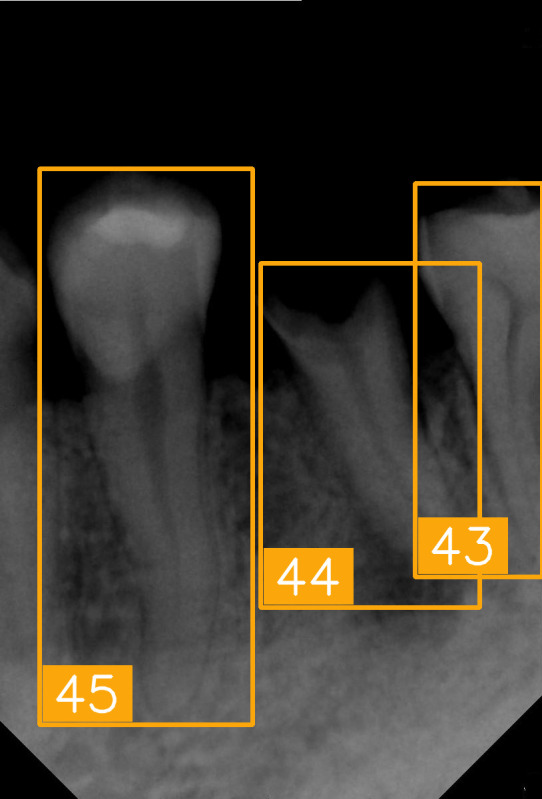	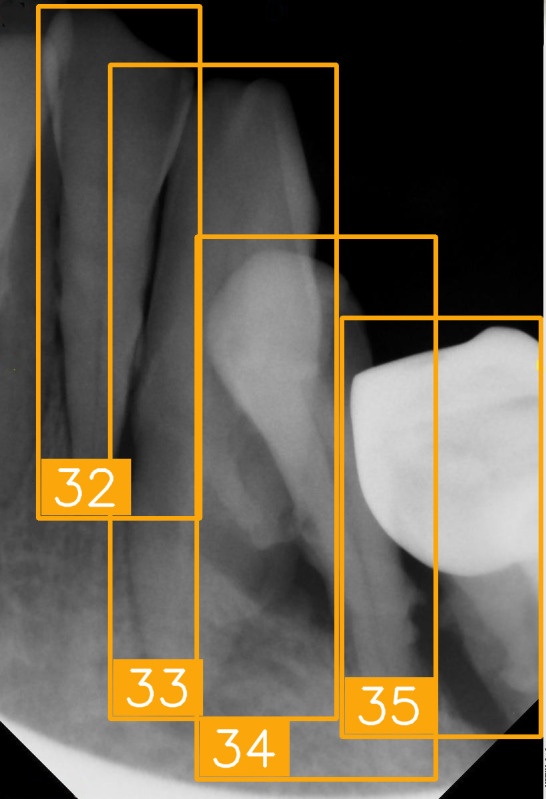	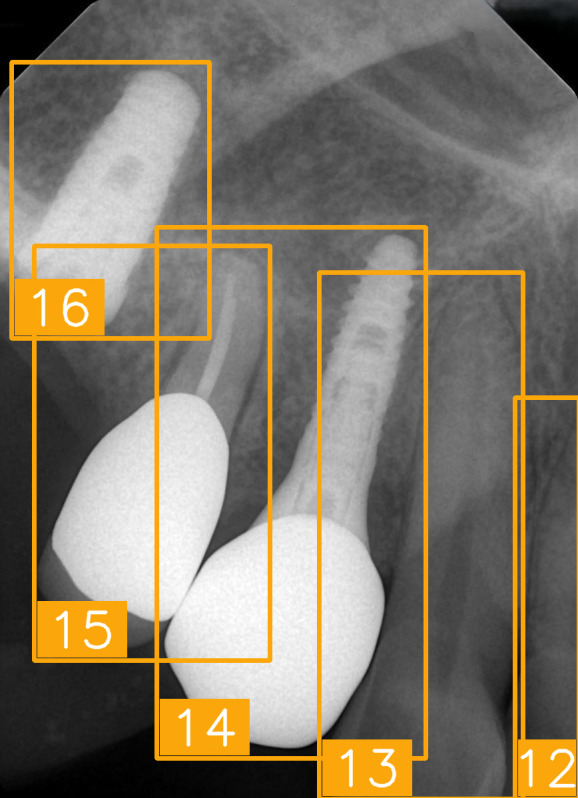	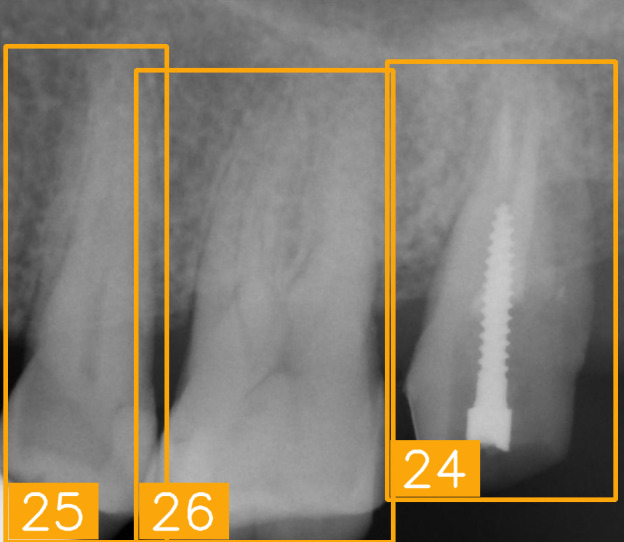
FDI ground truth number	45, 44, 43	32, 33, 34, 35	16, 15, 14, 13, 12	24, 25, 26
Inference Time	72.8 ms	77.2 ms	70.6 ms	72.4 ms

## Data Availability

The source code and dataset can be made available from the corresponding authors upon reasonable request (but only for research purposes).

## References

[1] Zardak N., Amini-Rarani M., Abdollahpour I., Eslamipour F., Tahani B. (2023). Utilization of dental care among adult populations: A scoping review of applied models. BMC Oral Health.

[2] Kodama T., Ida Y., Miura H. (2020). A nationwide survey on working hours and working environment among hospital dentists in Japan. Int. J. Environ. Res. Public Health.

[3] Pitts N.B., Zero D.T., Marsh P.D., Ekstrand K., Weintraub J.A., Ramos-Gomez F., Tagami J., Twetman S., Tsakos G., Ismail A. (2017). Dental caries. Nat. Rev. Dis. Primers.

[4] Li P., Wang Z., Zhang J., Zhao X., Wang Y. (2019). Orthodontic treatment planning based on artificial neural networks. Sci. Rep..

[5] Chen C.-K., Huang J.-Y., Wu Y.-T., Chang Y.-C. (2018). Dental scaling decreases the risk of Parkinson’s disease: A nationwide population-based nested case-control study. Int. J. Environ. Res. Public Health.

[6] Esquivel J., Villarroel M., Tran D., Kee E., Bruggers K. (2020). The utilization of snap-on provisionals for dental veneers: From an analog to a digital approach. J. Esthet. Restor. Dent..

[7] Akintoye S.O., Greenberg M.S. (2014). Recurrent aphthous stomatitis. Dent. Clin. N. Am..

[8] Patel R., Gallagher J.E. (2024). Healthy ageing and oral health: Priority, policy and public health. BDJ Open.

[9] Hasnain M.A., Ali Z., Maqbool M.S., Aziz M. (2024). X-ray image analysis for dental disease: A deep learning approach using EfficientNets. VFAST Trans. Softw. Eng..

[10] Turosz N., Chęcińska K., Chęciński M., Rutański I., Sielski M., Sikora M. (2024). Oral health status and treatment needs based on artificial intelligence (AI) dental panoramic radiograph (DPR) analysis: A cross-sectional study. J. Clin. Med..

[11] Meusburger T., Wülk A., Kessler A., Heck K., Hickel R., Dujic H., Kühnisch J. (2023). The detection of dental pathologies on periapical radiographs—Results from a reliability study. J. Clin. Med..

[12] Shehabeldin R.R., Hamama H.H. (2024). Introduction of ‘qpdb’ teeth numbering system. Heliyon.

[13] Rajendra Santosh A.B., Jones T. (2024). Enhancing precision: Proposed revision of FDI’s 2-digit dental numbering system. Int. Dent. J..

[14] Lin T.-J., Mao Y.-C., Lin Y.-J., Liang C.-H., He Y.-Q., Hsu Y.-C., Chen S.-L., Chen T.-Y., Chen C.-A., Li K.-C. (2024). Evaluation of the alveolar crest and cemento-enamel junction in periodontitis using object detection on periapical radiographs. Diagnostics.

[15] Wu P.-Y., Chen S.-L., Lin Y.-J., Wang L.-H., Chang Y.-C. (2024). Precision medicine for apical lesions and peri-endo combined lesions based on transfer learning using periapical radiographs. Bioengineering.

[16] Wang L.-H., Xie C.-X., Yang T., Tan H.-X., Fan M.-H., Kuo I.-C., Lee Z.-J., Chen T.-Y., Huang P.-C., Chen S.-L. (2024). Paper-recorded ECG digitization method with automatic reference voltage selection for telemonitoring and diagnosis. Diagnostics.

[17] Chen S.-L., Chen T.-Y., Huang Y.-C., Chen C.-A., Chou H.-S., Huang Y.-Y., Lin W.-C., Li T.-C., Yuan J.-J., Abu P.A.R. (2022). Missing teeth and restoration detection using dental panoramic radiography based on transfer learning with CNNs. IEEE Access.

[18] Lin Y.-J., Chen S.-L., Mao Y.-C., Chen T.-Y., Peng C.-H., Tsai T.-H., Li K.-C., Chen C.-A., Tu W.-C., Abu P.A.R. (2025). Precision oral medicine: A DPR segmentation and transfer learning approach for detecting third molar compress inferior alveolar nerve. IEEE J. Transl. Eng. Health Med..

[19] Chen S.-L., Chen T.-Y., Mao Y.-C., Lin S.-Y., Huang Y.-Y., Chen C.-A., Lin Y.-J., Hsu Y.-M., Li C.-A., Chiang W.-Y. (2022). Automated detection system based on convolution neural networks for retained root, endodontic treated teeth, and implant recognition on dental panoramic images. IEEE Sens. J..

[20] Lin Y.-J., Chen S.-L., Lu Y.-C., Lin X.-M., Mao Y.-C., Chen M.-Y., Yang C.-S., Chen T.-Y., Li K.-C., Tu W.-C. (2025). Deep learning-assisted diagnostic system: Implant brand detection using improved IB-YOLOv10 in periapical radiographs. Diagnostics.

[21] Chen I.-H., Lin C.-H., Lee M.-K., Chen T.-E., Lan T.-H., Chang C.-M., Tseng T.-Y., Wang T., Du J.-K. (2024). Convolutional-neural-network-based radiographs evaluation assisting in early diagnosis of the periodontal bone loss via periapical radiograph. J. Dent. Sci..

[22] Adnan N., Bin Khalid W., Umer F. (2023). An artificial intelligence model for instance segmentation and tooth numbering on orthopantomograms. Int. J. Comput. Dent..

[23] Ayhan B., Ayan E., Bayraktar Y. (2024). A novel deep learning-based perspective for tooth numbering and caries detection. Clin. Oral Investig..

[24] Ali M.A., Fujita D., Kobashi S. (2023). Teeth and prostheses detection in dental panoramic X-rays using CNN-based object detector and a priori knowledge-based algorithm. Sci. Rep..

[25] Sarsam W., Davies J., Al-Salehi S.K. (2025). The role of imaging in endodontics. Br. Dent. J..

[26] Görürgöz C., Orhan K., Bayrakdar I.S., Çelik Ö., Bilgir E., Odabaş A., Aslan A.F., Jagtap R. (2022). Performance of a convolutional neural network algorithm for tooth detection and numbering on periapical radiographs. Dentomaxillofac. Radiol..

[27] Chen C.-C., Wu Y.-F., Aung L.M., Lin J.C.-Y., Ngo S.T., Su J.-N., Lin Y.-M., Chang W.-J. (2023). Automatic recognition of teeth and periodontal bone loss measurement in digital radiographs using deep-learning artificial intelligence. J. Dent. Sci..

[28] Lee J.-H., Kim Y.-T., Lee J.-B. (2024). Identification of dental implant systems from low-quality and distorted dental radiographs using AI trained on a large multi-center dataset. Sci. Rep..

[29] Yaseen M. (2024). What is YOLOv8: An in-depth exploration of the internal features of the next-generation object detector. arXiv.

[30] Khanam R., Hussain M. (2024). YOLOv11: An overview of the key architectural enhancements. arXiv.

[31] Selvaraju R.R., Cogswell M., Das A., Vedantam R., Parikh D., Batra D. (2020). Grad-CAM: Visual Explanations from Deep Networks via Gradient-based Localization. Int. J. Comput. Vis..

